# Characteristics, treatment patterns, and outcomes of patients with metastatic prostate cancer during 2014–2022 in Pirkanmaa, Finland—an observational study

**DOI:** 10.1177/17562872261451289

**Published:** 2026-05-27

**Authors:** Mikko Moisander, Olivia Hölsä, Kaisa Teittinen, Kai Kysenius, Mikko Kosunen, Leena Lehmus, Teemu J. Murtola

**Affiliations:** Faculty of Medicine and Health Technology, Tampere University, Tampere, Finland; Department of Oncology, TAYS Cancer Centre, Tampere University Hospital, Tampere University, Tampere, Finland; Medaffcon Oy, Espoo, Finland; Pfizer Oy, Helsinki, Finland; Medaffcon Oy, Espoo, Finland; Pfizer Oy, Helsinki, Finland; Pfizer Oy, Helsinki, Finland; Faculty of Medicine and Health Technology, Tampere University, PO Box 100, Tampere 33014, Finland; Department of Urology, TAYS Cancer Centre, Tampere, Finland

**Keywords:** metastatic, observational study, prostate cancer, real-world data (RWD)

## Abstract

**Background:**

The prognosis of patients with metastatic prostate cancer (mPC) has changed profoundly in recent years. However, observational studies are required to understand the real-world outcomes of patients with mPC.

**Objectives:**

In this retrospective single-center observational study, we aimed to describe characteristics, treatments, and outcomes of patients with mPC in Pirkanmaa Hospital District (PHD), Finland, an area with a population of 0.5 million (10% of the Finnish population).

**Design:**

The incident patients with mPC treated in PHD during 2014–2022 were identified and stratified into patients with metastatic hormone-sensitive PC (mHSPC) and castration-resistant disease PC (mCRPC).

**Methods:**

Patients with mPC were identified from electronic healthcare data using diagnoses, treatments, procedures, and patient texts. Data on demographics, diagnoses, procedures, treatments, laboratory and pathology tests, and specialized healthcare contacts were collected from specialty care records. Due to changes in treatment practices over time, treatments and outcomes were analyzed in different time periods (mHSPC: 2014–2016 and 2017–2022, mCRPC: 2014–2017 and 2018–2022).

**Results:**

In total, 1083 incident patients with mPC were identified, of which 88% received treatment indicated for mPC. Out of the 795 patients with mHSPC, and 558 patients with mCRPC, 84% (*N* = 668) and 95% (*N* = 532) received treatment, respectively. The median overall survival (mOS) for all treated patients was 40.1 months. The mOS of treated patients with mHSPC increased from 32.6 months during 2014–2016 to 54.6 months during 2017–2022.

**Conclusion:**

Since the approval of enzalutamide as a first-line treatment for mCRPC, it has become the most common first-line treatment for mCRPC in PHD. Novel therapeutics, including docetaxel, enzalutamide, and abiraterone, have been adapted into clinical practice and are being used at earlier phases of the disease. Changes in treatment practices correlate with improved outcomes in patients with mHSPC in the PHD area.

**Trial registration:**

ClinicalTrials.gov NCT05701007.

## Plain language summary

Characteristics, treatments, and survival of men with metastatic prostate cancer in Pirkanmaa, Finland (2014–2022)

Prostate cancer is the most common cancer among men in Finland. While many cases are detected early and have a good prognosis, some men develop metastatic prostate cancer, meaning the cancer has spread beyond the prostate. In recent years, several new treatments have become available, but it is important to understand how these treatments work in everyday clinical practice outside clinical trials. In this study, we analyzed healthcare data from the Pirkanmaa Hospital District in Finland between 2014 and 2022. We identified 1,083 men with newly diagnosed metastatic prostate cancer and examined their clinical characteristics, treatments, and survival outcomes. Most patients (88%) received treatment intended for metastatic disease. We studied two groups separately: men whose cancer was still responsive to hormone therapy (metastatic hormone-sensitive prostate cancer) and men whose cancer had become resistant to hormone therapy (metastatic castration-resistant prostate cancer). Survival improved clearly over time for men with hormone-sensitive metastatic disease. The median overall survival increased from 32.6 months in 2014–2016 to 54.6 months in 2017–2022. The proportion of patients who died within the first three years after diagnosis decreased significantly. These improvements coincided with increased use of intensified treatment strategies, including chemotherapy and newer hormone-targeting medicines. In contrast, survival for men with castration-resistant metastatic disease remained relatively stable over the study period, despite wider use of modern therapies. Importantly, many older and frail patients received life-prolonging treatments in routine care, even though such patients are often underrepresented in clinical trials. These real-world findings suggest that treatment advances have improved outcomes for men with metastatic hormone-sensitive prostate cancer and provide valuable information for clinical decision-making and healthcare planning.

## Introduction

Prostate cancer (PC) is globally the second most common, and in most Western countries, including Finland, the most common cancer among men.^1,2^ Globally, the age-standardized incidence rate is 29.3 per 100,000, while in Europe it is 61.1 per 100,000.^3^ In 2023, the age-standardized incidence of PC in Finland was 79.8 per 100,000.^4^ The prognosis of PC is generally good. Patients with localized disease diagnosed at an early stage can have as high as 99% 5-year relative survival rate.^5^ PC often progresses slowly; however, up to one third of patients will later develop a metastatic disease, and more than 20% of patients present with regional or distant metastasis PC (mPC) at diagnosis.^5^

The treatment of PC has developed profoundly in recent years. Castration, induced by androgen deprivation therapy (ADT) that can be either medical or surgical, is the basis of treatment in advanced and/or metastatic disease. Although most patients respond initially to ADT, hormone-sensitive PC (HSPC) eventually develops into castration-resistant PC (CRPC). The disease can become metastatic either before or after the development of castration resistance.

Since the approval of docetaxel for mCRPC in 2004, outcomes for mCRPC have significantly improved.^6,7^ Moreover, docetaxel has improved the outcomes of patients with metastatic HSPC (mHSPC),^8^ and since 2016, it has been in use as a first-line treatment for mHSPC in combination with ADT in Finland.

The introduction of androgen receptor signaling inhibitors (ARSIs), including abiraterone, enzalutamide, and apalutamide, has expanded treatment options beyond chemotherapy. Abiraterone and enzalutamide were initially used as second-line treatment options for mCRPC; however, in 2018, both gained reimbursement as a first-line treatment option for mCRPC in Finland. Abiraterone or enzalutamide were not reimbursed for mHSPC during the study period.

In a clinical trial setting, many novel drugs have demonstrated significant increases in progression-free survival (PFS) and overall survival (OS). Existing evidence from observational studies is less comprehensive. Observational studies have suggested both undertreatment of mPC despite treatment availability,^9,10^ and differences between clinical trial and real-world populations.^11^

Elderly and frail patients are often not eligible for clinical trials, and thus, observational studies are required to fully understand the impact of novel drugs in routine clinical practice. Here, in this descriptive retrospective observational study, we utilized the healthcare registries of Finland and studied characteristics, treatment patterns, and outcomes of patients with mPC in Pirkanmaa Hospital District (PHD).

## Materials and methods

### Study design and data source

This study was a retrospective single-center study including incident patients with mPC (both mHSPC and mCRPC) during 2014–2022. Our data consisted of all patients with PC diagnosis (ICD-10-code C61*, *N* = 14,612) in Pirkanmaa hospital district (PHD) during 2007–2022 (2007–2013 being a washout period to exclude prevalent patients). PHD is an area with a population basis of 0.5 million (10% of the Finnish population), being one of the largest units treating PC in Finland. Patients with mPC were identified from electronic healthcare data using diagnoses, treatments, procedures, and patient texts. Data on demographics, diagnoses, procedures, treatments, laboratory and pathology tests, and specialized healthcare contacts were collected from specialty care records. Patients with mPC identified before 2014, patients living outside of PHD, or patients with other cancers other than PC were excluded. Analyses were carried out separately for patients with mHSPC and mCRPC when applicable. mHSPC index date was set as the detection date of mPC. For mCRPC, the index date was set when both mPC and CRPC had been detected, with the later date being the index date. Treatment patterns and outcomes were stratified by treatment lines and time windows based on the index date. Stratification time periods for patients with mHSPC were 2014–2016, and 2017–2022 and for patients with mCRPC 2014–2017 and 2018–2022 based on significant changes in the treatment landscape (introduction of docetaxel as a first-line treatment for mHSPC in 2016, and subsequently enzalutamide and abiraterone for first-line treatment of mCRPC in 2018). The study was conducted under permission from Pirha (research permission 1965/2023 R23501X). This study was conducted and reported in accordance with the Strengthening the Reporting of Observational Studies in Epidemiology (STROBE) guidelines for observational studies.^12^ A completed STROBE checklist is provided in the Supplemental Material.

### Patient identification

Patients with mPC (*N* = 2751) were identified from data using ICD-10 codes (C77*, C78*, or C79*; *N* = 1518; M49.5*C79.5, or M84.4; *N* = 49), tumor – node – metastasis (TNM) classification M1 (*N* = 374), record of treatment for mPC (abiraterone before 1.11.2021, apalutamide, cabazitaxel, docetaxel, enzalutamide before 1.11.2021, or radium-223; *N* = 752), records of radiation of metastasis (NOMESCO Classification of Surgical Procedures (Finland) procedure code WF049; *N* = 787), or “metastatic cancer” mentioned in patient texts (*N* = 2581). Patients with an mPC diagnosis before 2014, patients living outside of PHD, or patients with records of other cancers were excluded, resulting in 1083 incident mPC cases. Patients with CRPC were further identified based on laboratory records (⩾2 PSA measurements showing increase of 25% from PSA nadir and ⩾1 testosterone measurements of <1.73 nmol/L; *N* = 483) or a mention of “hormone-resistance” in patient texts (*N* = 626), or a record of treatment for CRPC (abiraterone, docetaxel before 1.1.2016, cabazitaxel, darolutamide, enzalutamide, radium-223, or lutetium-177; *N* = 681). Identification criteria of mPC and CRPC were defined in collaboration with clinical experts treating mPC at PHD to ensure accuracy in patient identification.

Patients with mPC were included in both analyses (mHSPC and mCRPC) if they were not CRPC at the mPC detection date but progressed to CRPC during the follow-up. Patients with mPC were followed up until the end of the study (31.12.2022), death, or moving outside of PHD. Progression to mCRPC from mHSPC was considered as a transition from the mHSPC cohort to the mCRPC cohort. 270 patients with mPC were included in both the mHSPC and mCRPC cohorts.

### Subgrouping

Treatment outcomes and patterns among patients with mCRPC treated with ARSIs and with the Eastern Cooperative Oncology Group (ECOG) performance status recorded were stratified by ECOG performance status (0–1, 2–4) and treatment (abiraterone, enzalutamide) as an exploratory analysis. According to the ECOG performance status classification, patients with ECOG 0–1 are classified as fully active/performance without restriction to restricted in physically strenuous activity but ambulatory and able to carry out work of a light or sedentary nature. Patients with ECOG 2–4 are classified from ambulatory and capable of self-care but unable to carry out work activities (ECOG 2) to disabled and unable to perform any self-care (ECOG 4).^13^

### Baseline and clinical characteristics

Baseline variables, including age, BMI, PSA, ALP, de novo metastatic (detection of mPC within 2 months from PC diagnosis), and ECOG performance status, were described (using the closest record up to 3 months from the index). Gleason score was reported as the closest record at any time from the index. Charlson’s comorbidity index (CCI)^14^ was reported from all recorded ICD-10 codes up to 5 years before the index (excluding ICD-10 codes C61*, C77*, C78*, and C79*). Additionally, clinical characteristics during the follow-up were reported, including length of follow-up, use of treatments indicated for mPC and mCRPC, procedures of palliative radiotherapy, use of strong opioids (morphine, oxycodone, fentanyl, methadone, hydromorphone), use of bone medication (denosumab, zoledronic acid), and records of symptomatic skeletal-related event (SSRE, ICD-10 M49.5*C79.5, M84.4 and S*2*).

### Treatment outcomes

Disease progression was defined as the time from mHSPC index (event-free state) to mCRPC index (event), death without progression to mCRPC (competing event), or end of the study (censoring event). Corresponding multistate Cox proportional-hazards models were fitted, including baseline clinical variables such as age, BMI, Gleason score, and de novo metastatic as covariates. OS was defined as the time from index until death (event) or end of the study (censoring event). Time to next treatment (TTNT) was defined as the time from initiation of the current treatment line until the initiation of the next treatment line (event), death (event), or end of the study (censoring event).

### Treatment lines

Treatment lines for patients with mCRPC were constructed from records of medication administrations and prescriptions after the index. Treatments included were ADTs (degarelix, goserelin, leuprorelin, triptorelin), docetaxel, cabazitaxel, apalutamide, bicalutamide, darolutamide, flutamide, Radium-223, Lutetium-177, zoledronic acid, denosumab, radiotherapy, abiraterone, and enzalutamide. Treatments were considered as combination therapies if the first records of multiple treatments were observed within 28 days. Otherwise, treatments were considered as monotherapies. Concomitant ADT was not considered as a combination therapy. ADT as a monotherapy (ADT only) was reported as a first treatment line if there were no records of other treatments within 28 days. Initiation of a treatment line was defined as the date of the first active substance record, and the end of a treatment line as an initiation of the next treatment line, end of follow-up (EOF), or a record of palliative care (ICD-10 Z51.5).

### Statistical analyses

Data was analyzed using primarily descriptive measures, including mean, standard deviation (SD), median, and interquartile range (IQR) values for continuous variables and proportions for categorical variables. The proportion of missing values was reported where applicable. For treatment outcomes, Kaplan-Meier estimates, corresponding time to event competing risk models, and Cox proportional-hazard models were utilized. *p*-Values and confidence intervals (CIs) were reported at a significance level of 95%. All statistical tests were descriptive in nature, and no adjustments for multiple comparisons were made. Structured data or structured variables (TNM classification, ECOG performance status, Gleason score, and radionuclide therapies (radium-223 and lutetium-177) extracted using text mining) were collected. Analyses were performed using R (version 4.3.3).^15^ Results with small patient numbers (1–4) were replaced by “<5” in results tables, and corresponding percentages were replaced by ‘-’ due to Finland’s authority (Findata) guidelines for secondary use of health data.^16^ Findata also ensured the anonymity of the results.

## Results

### Patients

A total of 1083 incident cases of patients with mPC were identified during 2014–2022 in the PHD region. Of all patients with mPC, 88% received treatment indicated for mPC. We further identified patients with mHSPC and mCRPC from this population. Out of the 795 patients with mHSPC, and 558 patients with mCRPC, 84% (*N* = 668) and 95% (*N* = 532) received treatment, respectively (Table 1).

**Table 1.** Baseline and clinical characteristics of treated patients with mHSPC and mCRPC in PHD area during 2014–2022.

Characteristic		mHSPC				mCRPC			
		*N* (%)	Median (IQR)		% missing	*N* (%)	Median (IQR)		% missing
Age (years)		668	72.7 (67.1–79.1)		0	532	74.9 (68.9–81.9)		0
BMI		189	26.7 (24.4–29.7)		71.7	142	26.5 (24.0–29.8)		73.3
PSA (ng/ml)		421	15.8 (3.7–73.0)		37.0	492	11.5 (3.7–40.9)		7.5
ALP (U/l)		267	93 (64.5–152.5)		60.0	437	86 (65–133)		17.9
Length of follow-up (years)*		668	1.3 (0.7–2.6)		0	532	1.6 (0.8–2.8)		0
De novo metastatic		406 (60.8)				231 (43.4)			
Treated for mCRPC*						435 (81.8)			
Treated for mPC**		668 (84.0)				532 (95.3)			
Palliative radiotherapy*		222 (33.2)				187 (35.2)			
SSRE*		30 (4.5)				42 (7.9)			
Bone medication*		154 (23.1)				292 (54.9)			
Opioid use*		248 (37.1)				321 (60.3)			
	Level	*N* (%)	Risk group	Total %	% missing	*N* (%)	Risk group	Total %	% missing
Gleason Score	3–5***	7 (1.1)	low/very low	5.8	5.2	11 (2.2)	Low/very low	7.9	5.1
	6	30 (4.7)				29 (5.7)			
	7	137 (21.6)	intermediate	21.6		86 (17)	Intermediate	17	
	8	87 (13.7)	high/very high	72.5		68 (13.5)	High/very high	75.1	
	9	329 (52.0)				274 (54.3)			
	10	43 (6.8)				37 (7.3)			
CCI	0	477 (71.4)				316 (59.4)			
	1	121 (18.1)				135 (25.4)			
	2	44 (6.6)				48 (9)			
	3	16 (2.4)				24 (4.5)			
	4+***	10 (1.5)				9 (1.7)			
ECOG performance status	0	165 (39.8)			37.9	97 (32.6)			44.0
	1	163 (39.3)				111 (37.2)			
	2	52 (12.5)				66 (22.1)			
	3–4***	35 (8.4)				24 (8.1)			

* Records during the follow-up (other variables reported as baseline values at diagnosis).

** Records during the follow-up and % of both treated and non-treated patients.

*** Patient groups combined to ensure patient anonymity.

ALP, alkaline phosphatase; BMI, body mass index; Bone medication (denosumab, zoledronic acid); CCI, Charlson comorbidity index; ECOG, Eastern Cooperative Oncology Group; mCRPC, metastatic castration resistant PC; mHSPC, metastatic hormone-sensitive PC; Opioid use (morphine, oxycodone, fentanyl, methadone, hydromorphone); Palliative radiotherapy (NOMESCO Classification of Surgical Procedures (Finland) procedure code WF049); PC, prostate cancer; PHD, Pirkanmaa Hospital District; PSA, prostate-specific antigen; SSRE, Symptomatic skeletal-related event (ICD-10 M49.5*C79.5, M84.4 and S*2*).

The median age of patients with mHSPC was 72.7 years and for patients with mCRPC it was 74.9 years. A total of 61% of patients with mHSPC and 43% of patients with mCRPC were de novo metastatic at PC diagnosis. Symptomatic skeletal-related events and the use of bone-protective medication, including denosumab and zoledronic acid, were more common among patients with mCRPC. Opioids were used more frequently in patients with mCRPC (60%) than in patients with mHSPC (37%). Additionally, 41% of patients with mCRPC and 29% of patients with mHSPC had a Charlson’s comorbidity index ⩾1. Majority (over 70%) of both mHSPC and mCRPC patients presented with a high-risk cancer, characterized by a Gleason score of 8–10.

When comparing the time-stratified populations of mHSPC (2014–2016 and 2017–2022) and mCRPC (2014–2017 and 2018–2022), we found that for mHSPC, the proportion of patients with de novo metastatic disease at PC diagnosis was higher in later time period (2017–2022), although the increase was not statistically significant. Conversely, the proportion of patients who received bone medication or opioids was significantly smaller in later period compared to earlier time period (2014–2016; Supplemental Table S1). For patients with mCRPC, the proportion of de novo metastatic cases was significantly higher in later time period (2018–2022). In contrast, baseline PSA levels and the proportion of patients who had received palliative radiotherapy, bone medication, and opioids were significantly smaller compared to earlier time period (2014–2017; Supplemental Table S2).

**Figure 1.**
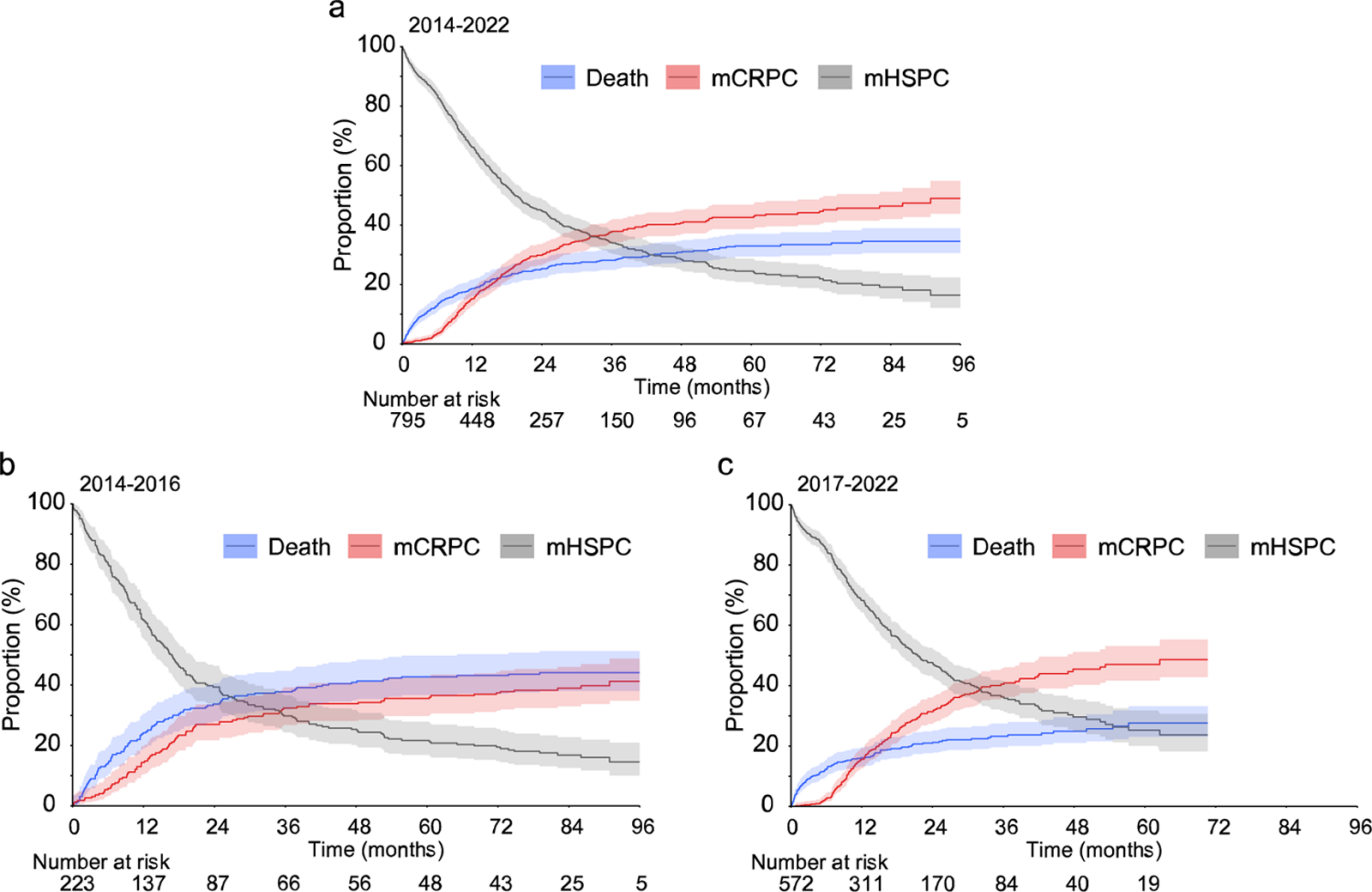
Competing risk curves for time to disease progression. The competing risk curves for patients with mHSPC (grey line), progression to mCRPC (red line) or death (blue line) with 95% CIs during (a) 2014–2022, (b) 2014–2016 and (c) 2017–2022. The grey line represents an event-free state (i.e., patients who have not progressed to mCRPC/death). The number at risk indicates the number of patients with mHSPC at different time points. mCRPC, metastatic castration resistant PC; mHSPC, metastatic hormone-sensitive PC; PC, prostate cancer.

The disease progression to mCRPC or death from an event-free mHSPC state was estimated. The median time to disease progression was 19.4 months (95% CI: 17.3–22.0; Figure 1(a)). During the first 3 years of follow-up, the progression to mCRPC was more likely than death with 38% progressing to mCRPC, and 28% to death before progression to mCRPC (Figure 1(a), Supplemental Table S3). The rate of progression was slower, although not statistically significant, in later time period of 2017–2022. The median time to disease progression was 16.8 months (95% CI: 13.5–19.2) during 2014–2016 (Figure 1(b), Supplemental Table S4), whereas during 2017–2022 it was 21.5 months (95% CI: 18.6–25.6; Figure 1(c), Supplemental Table S5). When comparing the disease progression during the first 3 years of follow-up for patients with mHSPC diagnosed in 2014–2016 versus 2017–2022, there was no statistically significant difference in the progression to mCRPC (32%; 95% CI: 27–39, vs 41%; 95% CI: 36–46, *p* = 0.6). However, the proportion of patients who died before progression to mCRPC during the first 3 years of follow-up was significantly lower in 2017–2022 when compared to earlier time period (38%; 95% CI: 32–45 vs 23%; 95 CI: 20–27, *p* < 0.001).

### Risk factors for disease progression

Cox proportional hazard models of disease progression (Supplemental Figure S1) indicated that older age and CCI score ⩾ 1 were associated with an increased risk of death (Supplemental Figure S1(a)). De novo metastatic disease or a high Gleason score (9–10) were identified as risk factors for progression to mCRPC, whereas Gleason score 7–8 was associated with decreased risk of death (Supplemental Figure S1(a)). Within time-stratified populations, older age was associated with an increased risk of death in both time periods. Patients with a high Gleason score were more likely to progress into mCRPC and less likely to die during the 2017–2022 period. A high CCI score was associated with an increased risk of progression to mCRPC during 2014–2016, while in 2017–2022, a high CCI score was associated with an increased risk of death (Supplemental Figure S1(b) and (c)).

### Survival

**Figure 2.**
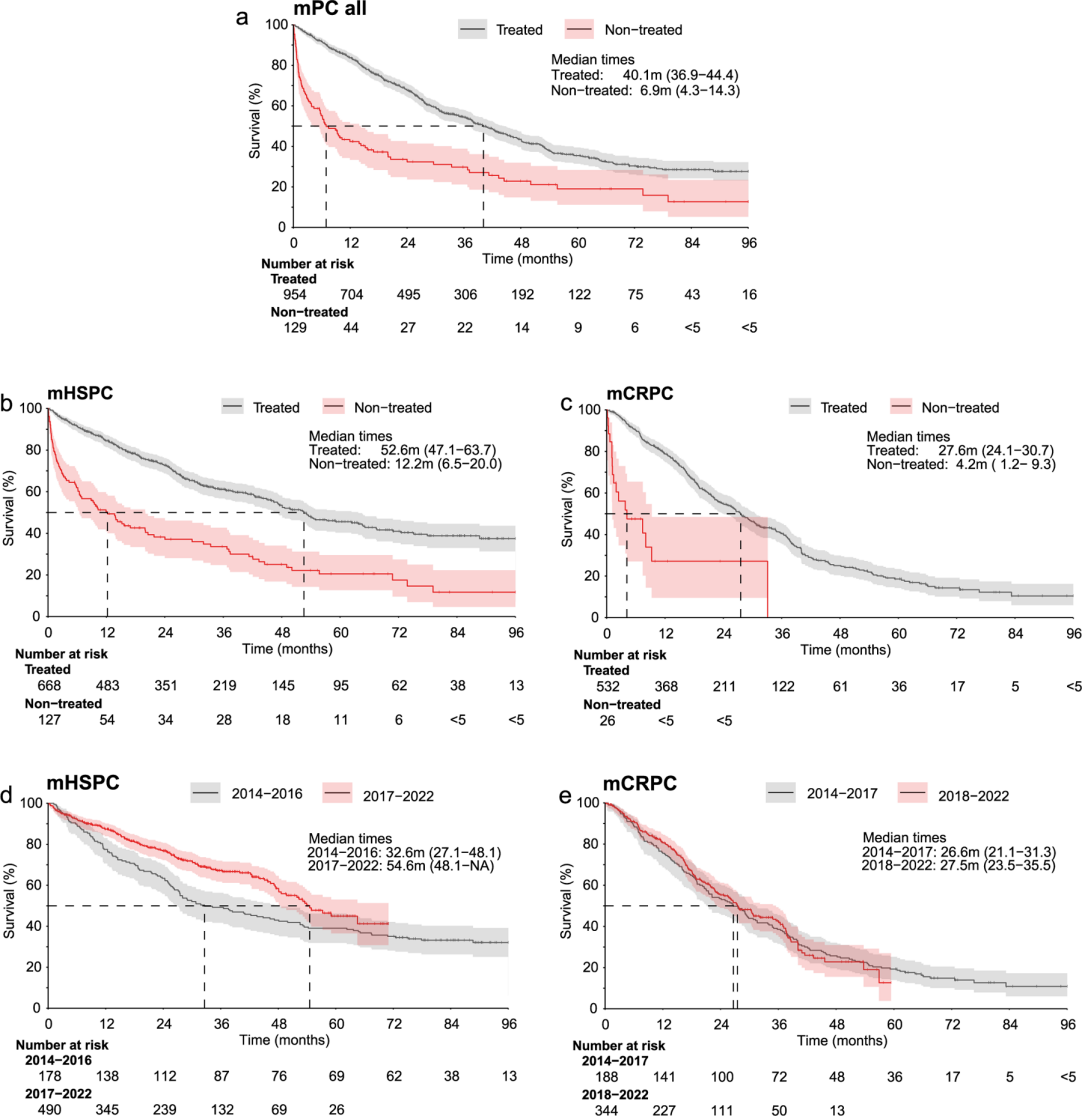
The OS of patients with mPC during 2014–2022. The OS of all treated and non-treated patients with (a) mPC during 2014–2022, (b) mHSPC during 2014–2016 and 2017–2022, and (c) mCRPC during 2014–2017 and 2018–2022. The OS of treated patients with (d) mHSPC during 2014–2016 and 2017–2022, and (e) mCRPC during 2014–2017 and 2018–2022. The OS was determined from the time of diagnosis. 95% CIs and median times are presented. mCRPC, metastatic castration resistant PC; mHSPC, metastatic hormone-sensitive PC; OS, overall survival; PC, prostate cancer.

The median OS (mOS, determined from index date) for all treated patients with mPC during 2014–2022 was 40.1 months and for untreated patients with mPC 6.9 months (Figure 2(a)). The mOS of treated patients with mHSPC was 52.6 months for the entire follow-up time (Figure 2(b)) and increased from 32.6 months during 2014–2016, to 54.6 months during 2017–2022 (Figure 2(d)). The mOS of treated patients with mCRPC was 27.6 months (Figure 2(c)). Surprisingly, the mOS of patients with mCRPC remained practically unchanged between 2014 and 2017 (26.6 months) and 2018–2022 (27.5 months; Figure 2(e)).

### Treatment evolution

**Figure 3.**
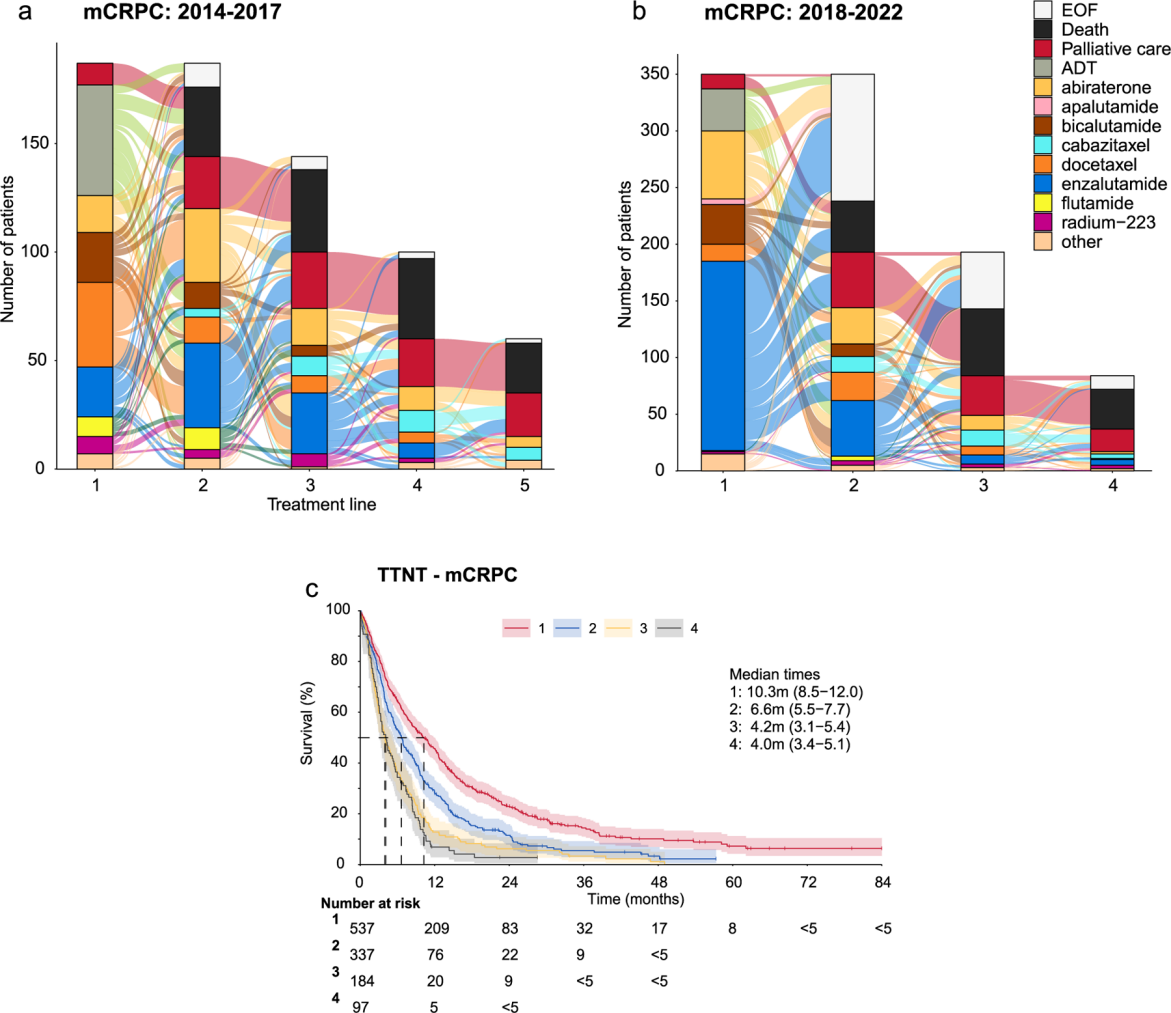
Treatment lines and TTNT in patients with mCRPC during 2014–2022. Treatment lines visualized by Sankey plots for patients with mCRPC during (a) 2014–2017 and (b) 2018–2022. (c) TTNT in patients with mCRPC during the entire follow-up period of 2014–2022 stratified by treatment lines (1–4). 95% CIs and median times are presented. EOF, End of follow-up; mCRPC, metastatic castration resistant PC; PC, prostate cancer; TTNT, Time to next treatment.

We next looked at treatments of mHSPC and mCRPC during different time periods (Figure 3(a)–(c), Supplemental Tables S6–S8). The most commonly used ADTs for treating mHSPC were degarelix and leuprorelin; only 10 (1.3%) patients were surgically castrated. Degarelix was received by 45%, and leuprorelin by 36% of patients with mHSPC during 2014–2022 (Supplemental Table S6). The use of degarelix increased from 27% in 2014–2016 to 52% in 2017–2022. While the use of bicalutamide decreased from 38% to 12% between 2014–2016 and 2017–2022, the use of docetaxel increased from 9% to 23%. During 2017–2022, 11% of patients with mHSPC received apalutamide, the only ARSI available for mHSPC in Finland during the study period.

A total of 80% of patients with mCRPC received ARSIs during 2014–2022 (Supplemental Table S6). While the use of enzalutamide increased from 55% to 67%, the use of abiraterone decreased from 45% to 32% between 2014–2017 and 2018–2022. During 2014–2017, 29% of patients received ADT only as first-line treatment for mCRPC, whereas 22% of patients received docetaxel, 13% enzalutamide, and 13% bicalutamide (Figure 3(a), Supplemental Table S7).

During 2018–2022, enzalutamide was the most common first-line treatment with 50% of patients receiving the treatment, followed by abiraterone (18%), bicalutamide (10%), and ADT only (11%; Figure 3(b), Supplemental Table S8). Enzalutamide, abiraterone, and docetaxel were the most common second-line treatments for mCRPC, with 33% of patients receiving enzalutamide, 28% abiraterone, and 10% docetaxel in 2014–2017 and 34%, 22%, and 17%, respectively, in 2018–2022. The percentage of those who received palliative care decreased both among patients with mHSPC (18% vs 13%) and mCRPC (60% vs 37%) during the follow-up (Supplemental Table S6).

During the entire follow-up period of 2014–2022, for patients with mCRPC, the median TTNT (time to death or next treatment) after initiating the first treatment line was 10.3 months, and 6.6 months after initiating the second treatment line (Figure 3(c)).

**Figure 4.**
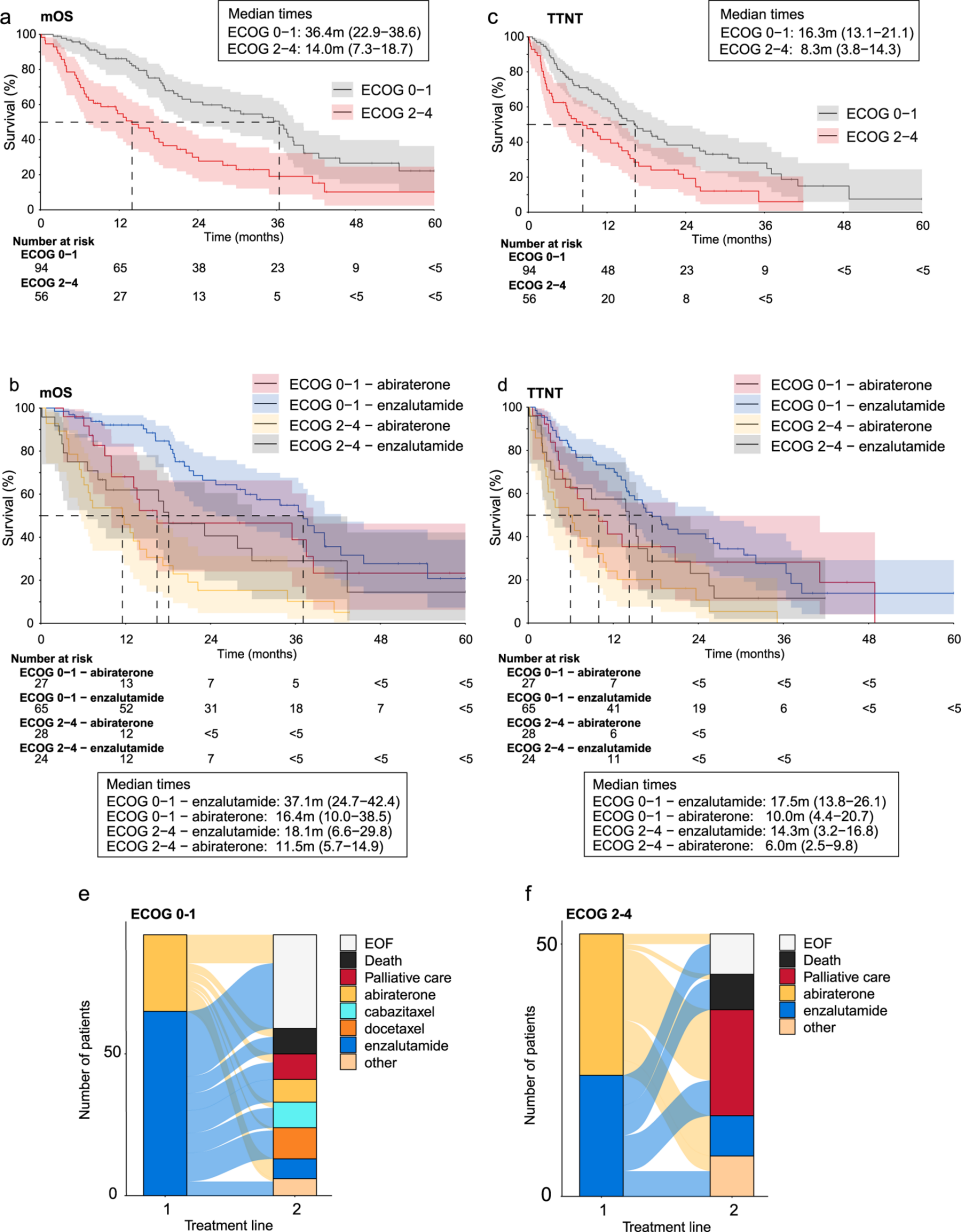
Treatment outcomes and treatment lines in patients with mCRPC and ECOG 0–1 or ECOG 2–4 with ARSIs as a first treatment line during 2014–2022. The mOS of patients with mCRPC and (a) ECOG 0–1 or ECOG 2–4, and (b) stratified by first line abiraterone or enzalutamide treatments. TTNT of patients with mCRPC and (c) ECOG 0–1 or ECOG 2–4, and (d) stratified by the first line abiraterone or enzalutamide treatments. Sankey plots describing treatment lines for patients with mCRPC and (e) ECOG 0–1 or (f) ECOG 2–4, and first line abiraterone or enzalutamide treatments. ECOG, Eastern Cooperative Oncology Group; mCRPC, metastatic castration resistant PC; mOS, median overall survival; PC, prostate cancer.

To shed light on the treatment of frail patients, we stratified the treatment outcomes of patients with mCRPC treated with ARSIs by the Eastern Cooperative Oncology Group (ECOG) performance status as an exploratory analysis. The mOS and TTNT of patients with ECOG 0–1 was longer when compared to patients with ECOG 2–4 (Figure 4(a) and (c)). The mOS and median TTNT of patients receiving abiraterone was inferior in both groups of ECOG 0–1 or ECOG 2–4 compared to patients receiving enzalutamide; however the difference was not statistically significant (Figure 4(b) and (d)). Patients with ECOG 0–1 received chemotherapy more often as a second treatment line, whereas patients with ECOG 2–4 were more often moved to palliative care (Figure 4(e)–(f)).

## Discussion

In this observational study, we show that the mOS of patients with mHSPC has increased significantly during recent years. Importantly, the 3-year death rate (deaths without progression to mCRPC) significantly decreased for patients with mHSPC.

Advancing age was identified as the most significant risk factor associated with increased risk of death without progression to mCRPC. Our findings aligned with previous reports indicating improvements in the survival over time, likely associated with developments in both treatment and lead-time due to more sensitive diagnostics.^17^

For patients with mCRPC, we report only a modest increase in mOS (from 26.6 months in 2014–2017 to 27.5 months in 2018–2022). The mOS of patients with mCRPC in this study was in line with a recent observational study from the US by Freedland et al.,^10^ which reported a mOS of 25.6 months for patients diagnosed with mCRPC between 2014 and 2019.

The improved prognosis can be attributed to recent advances in the treatment of mPC. Prior to the introduction of ARSIs, chemotherapy with docetaxel or cabazitaxel was considered as the only available treatment for patients with mCRPC. We found that the observed treatments and treatment lines for patients with mHSPC and mCRPC were consistent with local treatment guidelines, and new therapies have been introduced into practice as soon as they have received national reimbursement.

Another contributing factor to the observed outcomes could be the improved sensitivity for detecting metastatic disease compared to conventional imaging, particularly following the introduction of prostate-specific membrane antigen positron emission tomography (PSMA PET). This modality enables earlier detection of metastases and may therefore contribute to longer observed survival times through lead-time bias. However, PSMA PET was not routinely used for primary staging in PHD during the study period, and therefore, its overall impact on survival outcomes was likely limited.

Our real-world data indicates that ADT only was the most common first-line treatment for mCRPC during 2014–2017, suggesting a delay in the initiation of treatment of castration resistant disease. The proportion of patients receiving ADT only as a first-line treatment for mCRPC was substantially smaller in the later time period of 2018–2022, further suggesting an improvement in treatment initiation.

Moreover, observational studies have demonstrated that intensified treatment during hormone-sensitive phase of PC is associated with improved prognosis and treatment choices later during disease progression. For example, both clinical and observational studies have shown that upfront docetaxel or ARSI treatment in addition to ADT in mHSPC provides a survival benefit.^8,18–22^ A European study involving five countries found that patients who received ARSI for mHSPC mostly received taxane chemotherapy in mCRPC. Conversely, patients who had received taxane chemotherapy, or neither taxane chemotherapy nor ARSI in mHSPC, predominantly received ARSI in mCRPC.^23^ Recently, the introduction of triplet regimens in mHSPC, combining ADT, ARSI, and docetaxel, has expanded treatment options and is expected to further complicate treatment decisions in mCRPC.^24,25^

In our dataset, we observed a marked improvement in mOS among patients with mHSPC over time, whereas the corresponding change in the mOS of patients with mCRPC was only minor. When interpreting these trends, it is important to consider that both diagnostic and therapeutic developments can influence survival estimates. Earlier detection through improved imaging, particularly PSMA PET, can artificially extend measured survival due to lead-time bias.^26^ Additionally, treatment decisions during the mHSPC phase can affect subsequent mCRPC outcomes.^17,26^ Together, these factors highlight the importance of developing novel treatment options for mCRPC and for determining the optimal sequence of available treatments across mPC.

In this study, the mOS and TTNT of mCRPC patients receiving abiraterone as first treatment line were inferior in both groups of ECOG 0–1 or ECOG 2–4 compared to those receiving enzalutamide; however, the difference was not statistically significant. Consequently, conclusions cannot be drawn due to limited sample size, uneven distribution of treatments, high proportion of missing values in ECOG, and potential indication bias. Nonetheless, a similar trend has been observed in an observational study conducted by La et al.^27^

Previous observational studies have also reported significant undertreatment of mCRPC. An observational study conducted in Sweden from 2006 to 2016 found that only 36% of patients with mCRPC received life-prolonging treatment.^9^ By default, old and frail patients are often excluded from clinical trials due to concerns about the tolerability of new therapies. However, enzalutamide, for example, has been found to be well-tolerated in older patients.^28^ In our study, 78% of patients with mCRPC received life-prolonging treatment, which is consistent with findings reported by Freedland et al.^10^ We observed that ARSIs were also routinely used in patients with ECOG 2–4.

Since treatment practices vary regionally,^29^ observational study data from different countries are crucial for understanding global clinical practice, further supporting both clinical and health economic decision-making.

## Study strengths and limitations

This study is subject to limitations commonly associated with registry-based observational studies. The data reflect everyday clinical coding practices, which may be non-standardized and incomplete, and are subject to missing data or variations in coding practices.

This study utilized regional data from Finland, which may not fully represent the nationwide Finnish population. No sample size calculation was performed due to the retrospective study setting, which may result in limited statistical power for subgroup analyses, such as those stratified by ECOG status or treatment. Additionally, the method of patient identification favored specificity over sensitivity, and therefore, some untreated patients may not have been captured, introducing potential selection bias. Untreated patients who did not meet the mPC inclusion criteria likely had a poor prognosis, and therefore, their survival would be expected to be even worse than what is reported in this study. Although the inclusion criteria were developed in collaboration with clinicians to enhance accuracy, true identification accuracy remains unknown. Furthermore, due to the scope of the study and permissions, validation through clinical expert chart review was not feasible.

The strength of this study is the use of real-world data that has been collected from a single data source in a real-world setting, encompassing all diagnoses, procedures, and visits without stringent inclusion or exclusion criteria. In addition, Finland’s universal healthcare system, primarily funded by taxation, entitles all permanent residents in Finland, regardless of their financial situation, to the same level of public healthcare, and thus the data provides a representative sample of the population in the Pirkanmaa region, supporting the generalizability of these results to other regions in Finland.

## Conclusion

This observational study demonstrates that intensification of treatment for mHSPC has improved survival. However, it is important to note that the improvement may be influenced by lead-time bias. The data emerging from real-world treatment practices can provide valuable evidence to support clinical decision-making, particularly for patient subpopulations that are typically excluded from phase III clinical trials. This is exemplified by the use of ARSIs in the frail patients in our study.

## Supplemental Material

sj-docx-1-tau-10.1177_17562872261451289 – Supplemental material for Characteristics, treatment patterns, and outcomes of patients with metastatic prostate cancer during 2014–2022 in Pirkanmaa, Finland—an observational study Supplemental material, sj-docx-1-tau-10.1177_17562872261451289 for Characteristics, treatment patterns, and outcomes of patients with metastatic prostate cancer during 2014–2022 in Pirkanmaa, Finland—an observational study by Mikko Moisander, Olivia Hölsä, Kaisa Teittinen, Kai Kysenius, Mikko Kosunen, Leena Lehmus and Teemu J. Murtola in Therapeutic Advances in Urology

sj-xlsx-2-tau-10.1177_17562872261451289 – Supplemental material for Characteristics, treatment patterns, and outcomes of patients with metastatic prostate cancer during 2014–2022 in Pirkanmaa, Finland—an observational study Supplemental material, sj-xlsx-2-tau-10.1177_17562872261451289 for Characteristics, treatment patterns, and outcomes of patients with metastatic prostate cancer during 2014–2022 in Pirkanmaa, Finland—an observational study by Mikko Moisander, Olivia Hölsä, Kaisa Teittinen, Kai Kysenius, Mikko Kosunen, Leena Lehmus and Teemu J. Murtola in Therapeutic Advances in Urology

## Declarations

**Ethics approval and consent to participate:** The study was conducted under permission from the Research Ethics Committee of the Wellbeing Services County of Pirkanmaa (research permission 1965/2023 R23501X), in accordance with the Act on Secondary Use of Health and Social Data in Finland. According to the Finnish Act on Secondary use of health and social data (552/2019), no patient consent was required.

**Consent for publication:** Not applicable.

**Availability of data and materials:** The data used in this study is not publicly available and has been accessed through the study permit at the Wellbeing County of Pirkanmaa. The people named in the study permit have access to the data. The data has been pseudonymized to exclude names and other personal details of the patients by TAYS Research Services. Only the registry holders can grant rights to third parties to use the data in accordance with the Act on Secondary Use of Health and Social Data.
